# “Doctor, Can I Drink an Alcohol-Free Beer?” Low-Alcohol and Alcohol-Free Drinks in People with Heavy Drinking or Alcohol Use Disorders: Systematic Review of the Literature

**DOI:** 10.3390/nu14193925

**Published:** 2022-09-22

**Authors:** Elsa Caballeria, Maria Teresa Pons-Cabrera, Mercedes Balcells-Oliveró, Fleur Braddick, Rebecca Gordon, Antoni Gual, Silvia Matrai, Hugo López-Pelayo

**Affiliations:** 1 Addictions Unit, Psychiatry Department, Hospital Clínic; Villarroel 170, 08036 Barcelona, Spain; 2 Grup de Recerca en Addicions Clínic, Institut d’Investigacions Biomèdiques August Pi Sunyer (IDIBAPS); Rosselló, 149-153, 08036 Barcelona, Spain; 3 Red de Trastornos Adictivos, Instituto Carlos III; Sinesio Delgado, 4, 28029 Madrid, Spain

**Keywords:** low alcohol, no alcohol, NoLo, alcohol free, alcohol use disorder, heavy drinking, ethanol reduction

## Abstract

No- and low-alcohol drinks (NoLo) have been proposed as a potential way forward for the reduction in the alcohol burden of disease. So far, there is scarce synthesized evidence on the effects of these products on people with alcohol use disorder (AUD), or with a heavy or high-risk drinking pattern. The aim of the present study is to systematically review the evidence of the use of NoLo drinks in these populations. A total of 4045 records were screened and 10 studies were included in the review. Craving and desire to drink have been found to increase after the consumption of NoLo drinks in patients with AUD. The increase in craving correlates with the severity of alcohol dependence. In addition, in this population, alcohol-related cues might trigger physiological responses similar to those experienced when using alcohol. Furthermore, as mentioned, in some of the studies, consumption was shown to increase as the %ABV or verbal descriptors indicate lower alcohol. Last, according to the epidemiological data, heavy drinkers tend to use NoLo drinks on top of their usual alcohol consumption rather than as part of regular drinking patterns. Further studies should be conducted in people with AUD or people with a high-risk drinking pattern to provide new insight to guide clinicians, patients, and other stakeholders to make evidence-based informed decisions.

## 1. Introduction

Alcohol use is a major contributor to the burden of disease and mortality, and the seventh leading risk factor for both death and disability-adjusted life years (DALYs) [1]. Clear dose–response relationships to disease and to most alcohol-related health harms have been described, with a large percentage of alcohol-attributable net mortality burden (>77%) and overall mortality burden (67%) being due to heavy drinking (HD) [2]; defined as >60 g/day for men, and >40 g/day for women [3]. 

Notably, people with very high-risk drinking levels (VHRDL, >100 g/day for men and >60 g/day for women [3]) experience a disproportionate burden of disease and mortality [4], representing 54% of all cirrhosis cases, 44% of all pancreatitis cases, and 41% of all oral cavity cancers [4,5]. Their life expectancy is 21 to 35 years less than the general population [5]. Supporting heavy and risky drinkers to reduce their consumption can contribute significantly to reducing alcohol-related harm, both to individual drinkers and to society.

In this context, WHO’s global strategy to reduce the harmful use of alcohol encourages the alcohol industry to consider effective ways to contribute to reducing alcohol-related harm, in its core role as developer, producer, distributor, marketer, and seller of alcoholic beverages [6]. The stronger promotion of products with a lower alcohol concentration has been proposed as a desirable strategy (Anderson et al., 2020; WHO, 2010) [6]. However, the role of no-alcohol or low-alcohol (up to 1.2% alcohol volume) (NoLo) drinks, and the potential risks of their industry-sponsored promotion, especially in clinical populations (alcohol use disorder, AUD) or those with past alcohol use disorder (AUD), is still unclear. 

Patients in treatment for AUD often ask their practitioners for advice regarding the use of NoLo drinks. The lack of evidence around their impact on this group makes it difficult for clinicians to provide useful advice to their patients with AUD, in recovery, or for risky drinkers who are considering consuming NoLo products.

Research has described the possible positive and negative impacts of NoLo drinks in terms of harm reduction for the general population [2,7,8]. The authors argue that these products could possibly contribute to drinkers moving away from stronger products, reducing the overall alcohol consumed; they could also possibly facilitate cutting back on alcohol without standing out (reducing stigma); and, drinkers may use these NoLo products, rather than stronger drinks, in certain high-risk situations, such as driving. However, some potential negative impacts have also been pointed out: the use of these drinks favors the normalization of drinking culture; its use could lead to relapse in those with alcohol dependence; their use can promote the ‘taste for alcohol’ in populations in which alcohol use should be avoided (i.e., those with liver disease, young people, or pregnant women) [7,8]. 

In summary, there is some evidence for the potential of NoLo products to contribute to reducing the large burden of disease associated with heavy drinking. However, there is little synthesized evidence so far on the effects of NoLo drinks on patients with AUD, and heavy or high-risk drinkers. To address this gap, the aim of this study is to systematically review the evidence on the impact on heavy and high-risk drinkers and people with AUD of consuming NoLo products. 

No limitations have been placed on the scope of the studies included (to include not only results on consumption, but also to review the potential neurophysiological processes involved). The types of products included are: alcohol-free, non-alcoholic, de-alcoholized, and low-strength beers, ciders, wines, and spirits (sometimes referred to collectively as “NoLo drinks”). 

The research question is: “What advice can clinicians offer their patients with AUD, heavy or high-risk drinking, regarding NoLo products, based on the current scientific evidence?”

## 2. Materials and Methods

Data for the systematic review were collected following the PRISMA guidelines [9].

### 2.1. Search Strategy

We performed electronic searches in PubMed and Web of Science (WOS). A combination of the following terms was used: (‘Low alcohol’ OR ‘No alcohol’ OR ‘Zero alcohol’ OR ‘Alcohol-free’ OR ‘light’ OR ‘nolo’ OR ‘no-lo’ OR ‘mocktail’ OR ‘Alcohol free’ OR ((Reformulation OR Reduc*) AND (ethanol content)) OR (Reduc* ethanol strength) OR (Reduc* AND ‘alcohol strength’) OR (Reduc* AND (‘alcohol content’ or ‘ethanol content’))’ OR ‘Low strength alcohol’ OR ‘non-alcoholic’) AND (beer or cider or wine or spirits or ‘ready to drink’ or ‘fortified wine’ or ‘fermented beverages’ or ‘intermediate products’) AND (‘alcohol use disorder’ or ‘alcohol dependence’ or ‘alcohol abuse’ OR ‘problematic drinking’ OR ‘harmful drinking’ OR ‘risky drinking’ OR ‘heavy drinking’).

No date limits were set, so all of the relevant publications could be identified, and all of the articles published up to May 2022 were included. In addition, the included publications were revised to add studies that might be relevant but did not show up on the searches.

### 2.2. Selection Criteria

**Figure 1:**
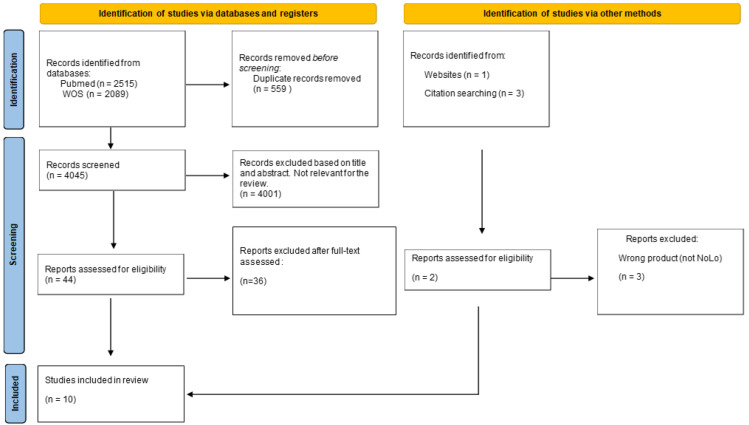
Flow diagram of included studies.

The search resulted in 4045 published articles (see Figure 1). We carried out an initial screening of the studies that appeared in the search, and studies were included if they met the following criteria: (1) At least one of the experimental conditions included the presentation of a placebo NoLo drink that has the same characteristics as the real drink; (2) the study sample included participants with a heavy drinking pattern or with AUD (and results were reported separately for these participants); (3) the outcomes of the study were related to alcohol use (craving, alcohol consumption), physiological responses to NoLo drinks, or the demographics of NoLo consumption. We excluded animal studies. Four additional studies were found from other websites or from cross-references. From the 4049 resulting articles, 10 met all of the inclusion criteria and were included for review.

### 2.3. Data Extraction

Two reviewers (MP, EC) extracted data independently and decisions regarding inclusion/exclusion were made based on consensus. In cases of disagreement, a decision was made by a senior researcher (HLP). The following information was extracted from the included articles: authors, year of publication, study design, sample characteristics, intervention, comparison or alternative intervention, outcome measure, and primary and secondary results. 

## 3. Results

After screening the titles and abstracts of 4045 articles, 45 full texts were assessed for eligibility. Of these, eight studies, and two more studies obtained from other websites or cross-references were eligible for systematic review (Figure 1). A summary of the main conclusions from the studies can be found in Table 1, and a description of the studies and main results is displayed in Table 2. 

### 3.1. Clinical and Experimental Studies

#### 3.1.1. Attitudes towards NoLo Drinks and Craving Following Their Use

Long et al. (1989) [10] explored the attitudes around low-alcohol drinks as well as their effects on craving among 31 inpatients and 67 former patients with risky drinking patterns that received an educational group session regarding the advantages and disadvantages of using low-alcohol drinks. Participants were offered several drinks to taste. The attitudes towards these drinks and craving for alcohol were assessed before and after the session. After six months, the use of low-alcohol drinks was assessed to explore its role in helping maintain post-treatment goals. Soft sparkling drinks were offered after an educational session on problem drinking as control condition. Craving was significantly raised over baseline after the session on low-alcohol drinks (r = 3.3, d.f. = 30, *p* > 0.01) with no differences between those who tasted the drinks and those who did not (Chi square = 0.023, d.f. = 1, *p* > 0.05). This increase was not observed after tasting the soft drinks. The severity of alcohol dependence was correlated to an increase over baseline for post-session rating of craving (Rho = 0.384, N = 20, *p* < 0.05). Low drinks were perceived as weak, unimportant, and non-habit forming. These were used by half of the former drinkers and viewed as a good alternative drink to blend in at social events. 

#### 3.1.2. Autonomic Reactivity to Alcohol-Related Cues

Two studies explored the autonomic reactivity to alcohol-related cues and included a placebo low-alcohol drink [11,12]. Sixteen patients with AUD [11,12] and sixteen social drinkers [12] were asked to hold a drink (either a 4–5% ABV beer or a non-alcoholic malt beer), while physiological measurements (heart rate and skin conductance) were taken. Later, they were asked to rate their desire to drink, and belief in whether the drink contained alcohol or not, were then instructed to drink, and all of the measurements were taken again. The procedure was repeated with an identical drink, and participants could get a third beer or a lottery ticket after completing an operant task. The patients with AUD receiving a real beer had a significantly higher desire to drink after trying the first drink, and patients with AUD receiving the placebo and controls receiving real beer experienced significantly higher desire than controls receiving placebo [11]. Skin conductance level (SCL) was higher in the participants with AUD, reaching significance only in those receiving real beer. In the patients with AUD, the SCL response to the presentation of the drink correlated to the desire to drink. A positive correlation was found between SCL response prior to consuming the drink and the severity of alcohol dependence in the AUD group receiving the real beer (*p* < 0.06) [11]. The SCL change during the initial presentation of the beer stimulus was higher in the AUD patients that perceived an alcohol effect following consumption [12].

The potential implications of neuroendocrine activity were explored among eight males with alcohol dependence (DSM-III) and nine healthy controls [13,14] that were presented with a real beer which they had to hold and smell without tasting. Then, they were asked to taste a non-alcoholic beer (placebo) which they thought contained alcohol. Several measures were obtained during both conditions (plasma, insulin, glucagon, cortisol levels, testosterone and luteinizing hormone levels; skin conductance, heart rate and anxiety and desire to drink). The cortisol values were depressed in the participants with alcohol dependence from the second blood sample to the end of the study, and also showed significantly larger and more rapid glucose and insulin responses following the consumption of the placebo beer [13]. Plasma testosterone decreased in the experimental group during the presentation of the real beer and increased after trying the placebo beer [14]. The luteinizing hormone decreased from baseline to the second blood test in the control group (Meyer et al., 1990). In the experimental group, a decrease was observed during the presentation of the real beer [14].

The effect of drinking restraint (particularly temptation) on drinking outcomes (consumption, subjective intoxication, and blood alcohol concentration) was studied using a taste-rating task (TRT) among 132 males with a moderate or heavy drinking pattern [15]. Each participant was presented two identical beers and was told that the beer contained alcohol or not. This expectation would be crossed with the receipt of an alcoholic or non-alcoholic beer. The consumption of alcohol was positively related to the temptation to drink more, however, expecting alcohol was not a significant predictor of alcohol consumption during the TRT. The authors found an interaction between restriction and the expected beverage, with the participants high on restriction consuming more when they expected alcohol and less when they expected a non-alcoholic beverage, and vice versa in the case of the participants low on restriction. The self-monitoring of alcohol consumption was not found to have an impact on consumption. Both the expectation of receiving an alcoholic beverage and the actual receipt of an alcoholic beverage were significantly related to subjective ratings of intoxication.

### 3.2. Epidemiological Studies

We only identified one report regarding the characteristics of NoLo drinkers that included results on moderate to heavy drinkers [7]. The authors conducted a survey among a nationally representative sample of 2003 adults, and a sample of 1010 past and present drinkers of NoLo products. NoLo drinks were found to be more likely to be used by: males, people aged 18–34, those in higher income socioeconomic groups, those with children under 18 in the household, people with a moderate or heavy drinking patterns, and younger drinkers who were more likely to use NoLo drinks containing cannabidiol. Regarding the reasons for using these drinks, risky drinkers were more likely to use NoLo products in specific situations (such as driving) rather than as a way to cut back on alcohol consumption, and would use these in addition to existing consumption of alcoholic beverages [7]. 

### 3.3. Product Description/Labelling Studies 

Three studies have explored how verbal descriptors of alcohol strength impact on product appeal [16], and on the amount of drink consumed [17,18]. 

In a randomized controlled trial (RCT) [17], 264 participants were presented with three glasses of their preferred drink (beer or wine) that included a label with a verbal descriptor of alcoholic strength: “very low”, “low”, or no descriptor. The results showed a significant linear trend, whereby the ml of drink consumed increased as the label on the drink indicated lower alcohol strength (Lin = 0.71, SE = 0.30, *p* = 0.015, 95% CI [0.13, 1.30]). This tendency was also observed in the participants with a risky drinking pattern, whose alcohol consumption was higher than those without a risky drinking pattern (2.46, SE = 0.72, *p* = 0.001, 95% CI [1.00, 3.83]) [17]. The consumption was significantly greater (approximately 23% more volume) when a numerical descriptor was included in the label (% alcohol by volume) compared to the verbal descriptor only (“Super low”), independently of the presence of a high-risk drinking pattern, and product appeal did not differ within the different experimental conditions [18]. 

In a different study to further explore the effects of verbal and numeric descriptors of strength on product appeal, the results showed that the products with the verbal descriptors “low” and “super low” had significantly lower appeal, especially when combined with no %ABV or 0%ABV [16]. Most of the participants correctly identified or erred on the side of caution when estimating the %ABV and calories of the given drinks. The results from this study did not differ among heavy or social drinkers [16].

## 4. Discussion

The aim of the present systematic review was to examine the evidence on the impact of consumption of no- and low-alcohol (NoLo) drinks among heavy drinkers and people with AUD, in order to support clinicians in giving advice to their patients regarding consumption of these products. Although the evidence is scarce and heterogeneous, some conclusions relevant for clinicians can be drawn, and several gaps for future studies have been identified.

### 4.1. Clinical/Experimental Studies

The results from the present review indicate that physiological responses in clinical samples towards alcohol-related cues (including placebo drinks) were different than in controls. Physiological reactivity to alcohol-related stimuli correlated with the desire to drink in people with AUD. Thus, neuroendocrine and other physiological responses (such as skin conductance) in response to alcohol-related stimuli among AUD patients, highlight the multivariate nature of the biological/behavioral state associated with alcohol dependence [11,12,13,14].

Neuroimaging studies have shown that ventral striatal dopamine is released following the administration of taste cues (alcohol-flavored sprays) [19,20] or olfactory cues (whiskey and beer odors) [21], even without significant pharmacologic effects. It is of note that the dopamine response was strongest in subjects with a greater genetic risk for alcoholism [19,20].

Taking all this data together, alcohol-related cues (i.e., product appeal, its flavor or odor, regardless of alcohol content or intoxicating effects), might trigger physiological reactions and arousal similar to those which occur when drinking an alcoholic beverage. Although there are not sufficient data to draw conclusions about the effects of these physiological reactions on further consumption, these results suggest that NoLo drinks could stimulate the desire to drink in people with AUD. Therefore, caution should be taken when offering advice on the consumption of NoLo products. 

### 4.2. Epidemiological Studies

Few data records were found on the use of NoLo products by heavy drinkers or patients being treated for AUD. NoLo consumers have been identified as more likely to be male, aged 18 to 34, with higher incomes, and in more affluent households. Moreover, heavy drinkers are more likely to have consumed an alcohol-free drink in the previous 12 months than non-drinkers [7,22]. Although heavy drinkers stand to benefit the most from the health gains through reduced alcohol consumption, they are more likely to use them in addition to or as only occasional substitution to their current alcohol use rather than as a longer-term replacement [7,23]. On the other hand, among clinical samples [10], NoLo drinks have been reported as an acceptable alternative to alcoholic drinks at social events. Patients indicate that they would recommend them to other people trying to quit, and do not consider these products facilitators of relapse [10].

The use of NoLo drinks in low socioeconomic groups is limited; therefore, heavy drinkers with a higher socioeconomic level may have more access to these products as a tool to reduce ethanol consumption [22], and this could have implications for future health inequalities [7]. 

### 4.3. Product Description/Labelling Studies 

Labeling NoLo alcohol products by alcohol volume content or using verbal descriptors of alcoholic strength may lead to an increase in alcohol consumption [17,18], especially when alcoholic strength is represented using a numerical descriptor [16]. These results are in line with previous studies that suggest the percentage of alcohol presented in the label may be used as a reference to purchase stronger alcohol products [24,25] and thus serve to further increase heavy drinking [24], or pouring larger servings [26]. However, other studies suggest that the availability of NoLo beers seems to replace rather than being a gateway to purchasing same-branded higher strength beers [27]. 

The impact of labeling on consumption needs to be considered when applying policies aimed at encouraging consumers to switch to lower alcohol alternatives. For instance, reducing the price per drink while not highlighting the lower %ABV of the drink [18].

### 4.4. What Advice Can Clinicians Offer Their Patients with AUD, Heavy, or High-Risk Drinking, Regarding NoLo Products, Based on the Current Scientific Evidence?

In our opinion, despite the patients’ attitudes towards NoLo products being favorable and the idea that these products could be suitable in certain situations, the existing evidence forces us to be cautious when recommending NoLo drinks to clinical populations and people with risky drinking patterns. Craving has been found to increase after the consumption of NoLo drinks, and this increase correlates with the severity of alcohol dependence. In addition, alcohol-related cues might trigger physiological responses similar to those experienced when using alcohol, and the desire to drink could subsequently be increased. Furthermore, consumption has been shown to increase when using products marked as lower in alcohol concentration. Lastly, heavy drinkers tend to use NoLo drinks on top of their usual alcohol consumption, rather than as part of their regular drinking patterns. In summary, considering the available evidence, we consider that the precautionary principle should prevail. However, this principle needs to be adapted when applied to clinical practice, and healthcare professionals should prudently assess the benefits (beneficence principle) and risks (no maleficence principle), while respecting the patients’ autonomy [28,29,30].

### 4.5. Limitations

A series of limitations must be mentioned. First, the heterogeneity of the methodological approaches of the included studies, together with the low number of articles identified, hinders us from drawing firm conclusions. Although non-systematic searches were conducted in other databases (Scopus and the Cochrane Library) and the references in the included articles were revised, the systematic search was conducted only among two databases so relevant articles could have been missed. Moreover, almost all of the included studies were not designed to ascertain how low-alcohol options modify the consumption of ethanol and their associated harms. Nonetheless, some of the studies investigated the effect of low-alcohol options on cravings, anxiety, and desire to drink, which their consumption is known to impact on. This review focuses on a sub-population of heavy drinkers or clinical populations. Thus, while some of the studies provided disaggregate data to facilitate the understanding of the results, a number of the studies were not conducted specifically on at-risk individuals. Above all, no prospective clinical or naturalistic studies were found, which limits the evidence on consumption of alcohol and its related harms.

### 4.6. Gaps and Future Research

There is a gap in the study of epidemiology regarding the use of no- and low-alcohol beverages in AUD patients, and in samples with a heavy drinking pattern, to clarify whether the use of NoLo drinks substitutes or adds to the existing alcohol use in this specific sector of population. These studies need to consider the sociodemographic variables (i.e., socioeconomic status) that can facilitate or hinder the access to these products, as well as other confounding variables (i.e., family history of addiction) that could play a role in the pattern of use of these drinks. Second, clinical longitudinal studies would provide a greater understanding of the effects of these products in maintaining treatment goals (reduction in alcohol use, abstinence) and their effects on craving. Randomized controlled trials would provide the most useful insight if ethical and practical issues can be solved. Observational, prospective well-designed studies would be a good alternative. A conflict of interest with the alcohol industry should be avoided in this area of research. 

## 5. Conclusions

NoLo products are becoming increasingly available and popular as alternatives to traditional alcoholic beverages. However, there is not enough evidence to clearly guide advice regarding consumption of these products by people with AUD, heavy, and high-risk drinkers. Craving and desire to drink increase after the consumption of NoLo drinks, and consumption increases as the descriptors in the label indicate a lower ethanol concentration. 

This gap in the evidence should be filled urgently as it has important clinical implications for those who must deal with AUD (patients and practitioners) as well as in public health policies, especially in the field of publicity and taxes of this type of beverages. Policy areas interact over the issues around NoLo—clinical guidance, labelling, branding, and marketing, especially—and pricing seems to be key. Special considerations must be kept in mind for clinical sub-populations. We are still far from knowing the risks and potential benefits from NoLo products that would allow clinicians to discuss with the patient the usefulness of these drinks in the treatment plan. 

Randomized controlled trials, observational prospective studies, and laboratory studies could provide new insights to guide clinicians, patients, and other stakeholders to make evidence-based informed decisions. Furthermore, these decisions could vary depending on the patients’ characteristics and the evolutionary stage of the disorder and associated comorbidities. Remarkably, the alcohol industry might have interest in promoting NoLo drinks. Consequently, research in this area must be free of any conflict of interest. 

## Figures and Tables

**Table 1:** Summary of main conclusions.

Study	Main Conclusions
Clinical and Experimental	
Craving [10]	- Craving increased after offering NoLo drinks, with a significant correlation with severity of alcohol dependence. - NoLo drinks were viewed as a good alternative to blend in at social events (used by 47% of the ex-patients after leaving treatment).
Autonomic reactivity to alcohol-related cues [11,12]	- Desire to drink: significantly higher in AUD patients receiving a real beer; significantly higher in patients with AUD receiving placebo beer vs. controls receiving placebo. - Skin conductance level (SCL): ◦ Higher in AUD vs. controls, only significant in AUD patients receiving a real beer ◦ In AUD, correlation between SCL and the desire to drink ◦ In AUD, a significant change in SCL was observed when they perceived an alcohol effect after the consumption.
Neuroendocrine activity [13,14]	- Participants with AUD dependence: ◦ Depressed cortisol values ◦ Significantly larger and more rapid glucose and insulin responses to the consumption of NoLo beer ◦ Decreased plasma testosterone during the presentation of the real beer and increased after trying the placebo beer ◦ Luteinizing hormone decreased during the presentation of the real beer.
Drinking restraint (particularly temptation to drink) [15]	- Consumption was positively related to the temptation to drink - Interaction between restriction and the expected beverage: high restriction participants consumed more when they expected alcohol and less when a non-alcoholic beverage was expected, and vice versa for those on low restriction. - The expectation of receiving an alcoholic beverage and the actual receipt of it were significantly related to subjective ratings of intoxication.
Epidemiological Studies	
[7]	- Heavy drinkers tended to use NoLo drinks on top of the existing consumption of alcoholic drinks. - HD were more likely to use NoLo drinks on specific occasions rather than as a way to cut back alcohol consumption.
Product Description/Labelling Studies	
Product labeling [16,17,18]	- Significant linear trend whereby the ml of drink consumed increased as the label on the drink indicated lower alcohol strength. - Consumption was greater when a numerical descriptor of alcohol strength was included. - Product appeal decreased as the %ABV decreased. Products with the verbal descriptors “Low” and “Super low” had significantly lower appeal (especially if combined with no or 0% ABV).

ABV: Alcohol by volume; HD: Heavy drinkers.

**Table 2:** Summary of the included articles on the use of NoLo products in patients with AUD, heavy or high-risk drinking.

Author and Year	Study Design	Population	Intervention	Comparator	Outcome	**Results**
Long et al., 1989 [10]	Cross-over	31 inpatients and 67 former patients who had completed at least 1 week of a 5-week therapy program covering detoxification, education, skill training and relapse prevention strategies.	Educational group session in which the advantages and disadvantages of using low-alcohol drinks were discussed and in which they were offered to taste several drinks.	Soft drinks were offered after an educational session on problem drinking, to control the effects of convivial drinking.	Craving: at baseline (8 assessments during the weeks before and after the low-alcohol drink session), 1 h before, immediately before and immediately after the session Attitudes toward low-alcohol drinks: before and after the session. Use of the low-alcohol drinks to maintain post-treatment goals.	Craving was significantly raised over baseline (r = 3.3, d.f. = 30, *p* > 0.01) after the low-alcohol drinks session. No differences between subjects who had tested the low-alcohol drinks and those who had not (Chi square = 0.023, d.f. = 1, *p* >0.05). No increase in craving after the soft drink control session (t = 0.31, d.f= 19; *p* > 0.05). A significant correlation between severity of dependence and increase craving after sessions was found (Rho = 0.384, N = 20, *p* < 0.05). Attitudes toward low-alcohol drinks: INPATIENTS: 65% had used low alcohol drinks, and 19% of these have been using them daily, and 24% used them once a week. A total of 42% of the ones that used low drinks before felt that they helped to cope with the urge to drink alcohol. A total of 79% of the sample had a ‘favorable’ attitude toward low-alcohol drinks; 89% disagreed with the idea that low-alcohol drinks contributed to relapse. A total of 58% felt that low-alcohol drinks would be most acceptable alternatives to alcoholic drinks and were useful in helping them to join in at pubs and parties (47%). Of the inpatients, 55% would use the low-alcohol drinks after discharge; 50% would buy them in pubs and 42% at home. FORMER PATIENTS: A total of 47% had used low-alcohol drinks since leaving treatment (26% daily, 40% once/week). In all, 85% had a post-treatment goal of abstinence, 44% felt the use of low-alcohol drinks helped with this. Finally, 39% would recommend the use of these products to problem drinkers.
Kaplan et al., 1983 [11]	Double blind, placebo-controlled trial	16 alcoholic patients (with a history of heavy drinking of at least 5 years) and 16 control subjects (social drinkers)	Participants were randomly assigned on a double-blind basis to either an ethanol or placebo condition. First, they do not drink it, only hold it while measurements are taken. Then, they are asked about the desire to drink, and their belief of whether the drink contained alcohol. Second, An identical second drink was presented and consumed Third, Subjects were instructed that they had an opportunity to work for a third drink or another reward. They must perform an operant task to obtain the reward, after which they must choose the drink or the other reward. The withdrawal symptoms and drinking behavior on the previous month is previously examined.	Placebo beer (non-alcoholic malt beverage) that participants believe contains alcohol.	To investigate the contributions of subclinical withdrawal symptomatology in the previous 30 days to psychophysiological arousal, desire to drink, and operant behavior associated with alcohol within the clinical laboratory.	Alcoholics showed a greater desire to drink than controls. There was also a significant correlation between autonomic arousal and desire to drink among alcoholics but not controls. There was some evidence that arousal was related to alcohol dependence among alcoholics. Placebo responding among alcoholics was also related to alcohol dependence. Desire to drink, withdrawal symptomatology, and heart rate accounted for over 57% of the variance in predicting which alcoholics would choose the drink reward following the operant task.
Kaplan et al., 1984 [12]	Double blind, placebo-controlled trial	16 male alcoholic inpatients (with at least 5 years of alcohol dependence) undergoing alcohol treatment.	Skin conductance level is recorded during the presentation of a beer drink or placebo (randomly assigned) and subjects are asked if they thought they had just consumed an alcoholic drink.	Placebo beer (non-alcoholic malt beverage).	First, to describe the relationship between autonomic reactivity to an alcohol stimulus prior to the consumption, second, to describe the perceptions of an alcohol effect immediately following consumption of either real beer or placebo in alcoholic subjects.	SCL increases to the presentation of beer stimuli prior to consumption were highest among alcoholics who perceived the drink as ‘real beer’ following consumption. Perception of the drink as ‘real beer’ was not related to receiving real beer.
Vasiljevic et al., 2018 [17]	RCT	264 (132 weekly wine drinkers and 132 weekly beer drinkers)	Group 1: label displaying the verbal descriptor Super Low + 4% ABV for wine/1% ABV for beer; Group 2: verbal descriptor Low + 8% ABV for wine/3% ABV for beer	Group 3: no verbal descriptor of strength + the average ABV of 12.9% for wine/4.2% for beer.	Primary: Total volume of drink consumed (in ml). Secondary: product appeal, understanding of alcohol strength, calorie content, guilt related to consumption. Other measures: risky drinking, motivation to reduce consumption, self-licensing.	ml of alcohol consumed increased as the label on the drink denoted successively lower alcohol strength (Lin = 0.71, SE = 0.30, *p* = 0.015, 95% CI [0.13, 1.30]) Group 1 drank more (M = 213.77, SD = 124.05) vs. Group 3 (M = 176.85, SD = 116.41), BD2 = 1.43, SE = 0.61, *p* = 0.019, 95% CI [0.24, 2.61]. No differences between Groups 2 and 3 (BD1 = 0.59, SE = 0.63, *p* = 0.340, 95% CI [−0.66, 1.80]). Risky drinkers drank more than non-risky drinkers, (BD8 = 2.46, SE = 0.72, *p* = 0.001, 95% CI [1.00, 3.83]).
Vasiljevic et al., 2018 [16]	3 × 6 between-subjects, randomized study	1697 wine drinkers (41% with a risky drinking pattern); 1693 beer drinkers (55.9% risky drinking pattern)	18 groups with one of three levels of verbal descriptor (Low; Super Low; No verbal descriptor) and six levels of %ABV (five levels varying for wine and beer, and no level given.	Same as intervention.	Primary: product appeal. Secondary: Understanding of alcohol strength and calorie content.	Appeal decreased significantly as %ABV decreased with lowest appeal for wine with 0%ABV and 4%ABV, and for beer with 1%ABV and 2%ABV (*p* ≤ 0.001, for the comparison with Regular). Appeal for Low verbal descriptors was lowest when combined with No %ABV, and for Super Low appeal was lowest when combined with 0%ABV. Both Low and Super Low verbal descriptors had a similar detrimental impact on appeal (pswine < 0.001; psbeer < 0.002). Heavy drinking pattern did not affect the results.
Vasiljevic et al., 2021 [18]	RCT	147 weekly wine drinkers	Group 1: verbal descriptor only (Super Low). Group 2: numerical descriptor only (4% ABV); and Group 3: verbal and numerical descriptors combined (Super Low 4%ABV).	Same as intervention.	Primary: Total volume of drink consumed (mL). Secondary: product appeal.	Participants randomized to the numerical descriptor label group (4%ABV: M = 155.12 mL, B = 20.30; 95% CI = 3.92, 36.69; *p* value = 0.016) and combined verbal and numerical descriptor label group (Super Low 4%ABV: M = 154.59 mL, B = 20.68; 95% CI = 4.32, 37.04; *p* value = 0.014) drank significantly greater amounts than those randomized to the verbal descriptor label group (Super Low: M = 125.65 mL). Self-reported appeal of the wine did not differ between the three groups (all Ps > 0.082).
Dolinsky et al., 1987 [13]	Experimental	EG: 8 male inpatients with alcohol dependence (AD) (DSM-III) hospitalized from 7–14 days of a 21-day alcohol rehabilitation program. 9 control subjects	Smell a real beer and then drink a “placebo” beer they believed contained alcohol. Outcomes were assessed at: - Baseline - Baseline (after 45′) - During presentation of the drink - After having the drink - 60′ after having the drink.		Heart rate, skin conductance. Plasma insulin, glucagon, and cortisol levels.	Cortisol values were depressed in the EG and remained so throughout the study (group F(9,135) = 7.51, *p* = 0.05). The EG presented significantly larger and more rapid glucose and insulin responses than the CG following the consumption of the placebo beer, which they believed contained alcohol (group x time: glucose, F(9,135) = 2.28, *p* = 0.05; Insulin, F(9,135) = 3.5, *p* < 0.001). Both groups experienced an increase in desire to drink while smelling the real beer (time: F(3,45) = 5.14, *p* < 0.05). Heart rate was greater in the EG during the baseline assessments (B1:76 vs. 65; B2: 71 vs. 65, Group: F(1,14) = 5.45, *p* < 0.05).
Meyer et al., 1990 [14]	Experimental	EG: 8 male inpatients with alcohol dependence (AD) (DSM-III) hospitalized from 7–14 days of a 21-day alcohol rehabilitation program. 9 control subjects	Smell a real beer and then drink a “placebo” beer they believed contained alcohol.		Primary: Changes in plasma concentrations o of testosterone and Luteinizing Hormone (LH). Secondary: changes in subjective reports of anxiety and alcohol craving.	EG presented a decrease in plasma testosterone during the drink presentation period and an increase relative to the control group during the post-drink period (Group x Time: testosterone F = 4.18, 9/126 df, *p* < 0.001). For LH: controls showed a decrease relative to EG (Group x Time: LH F = 3.83, 1/14 df, *p* < 0.1). In the EG, LH decrease during the pre-drink period (while holding the beer) (Group x Time: LH F = 4.66, 1/15 df, *p* < 0.05). No association between Testosterone levels and secondary outcomes (at the pre-drink nor post-drink period).
Corfe et al., 2020 [7]	Report examining the “NoLo drinks” market in UK, based on desk research, consumer survey, and interviews with professionals.	The online survey, taken between the 29 May 2020 and the 4 June 2020, with two samples: A nationally representative sample of 2003 adults. A sample of 1010 past and present drinkers of NoLo products.			Exploring the role that NoLo products can play in improving public health outcomes, considering alcohol harm. On the report disaggregated data on moderate and heavy drinkers is offered.	Moderate and heavy drinkers appear more likely than non-drinkers and light drinkers to consume NoLo drinks on specific occasions. (such as when driving), and on top of (rather than instead of) consumption of stronger drinks. This might limit the potential health benefits that could be realized from increased use of NoLo products.
Collins et al., 1996 [15]	RCT (Balanced placebo design)	132 young males with a moderate to heavy drinking pattern.	30 min taste-rating task (TRT): patients’ expectations of receiving an alcoholic or a non-alcoholic beer were crossed with the receipt of an alcoholic or a non-alcoholic beer. Four groups: ENA/RNA; ENA/RA; EA/RNA; EA/RA. Taste characteristics of the beer.		Effects of drinking restraint (temptation and restriction), beverage instructions and content and self-monitoring in alcohol-related outcomes (consumption, subjective intoxication and blood alcohol concentration (BAC)).	Consumption during a 30-min taste-rating task was positively related to the temptation to drink (i.e., difficulty controlling alcohol intake, drinking in response to negative emotions. There was also an interaction between restriction (an aspect of restraint) and expected beverage with high restriction subjects tending to drink more when they expected alcohol.

RCT: Randomized controlled trial; ABV: Alcohol by volume; LH: Luteinizing Hormone; AD: Alcohol dependence; NoLo: no alcohol or low alcohol; TRT: Taste-rating task; BAC: Blood alcohol concentration; ENA: Expect no alcohol; RNA: receive no alcohol; RA: receive alcohol; EA: expect alcohol.

## Data Availability

All the data has been provided in the main text.
